# Immune classification and identification of prognostic genes for uveal melanoma based on six immune cell signatures

**DOI:** 10.1038/s41598-021-01627-2

**Published:** 2021-11-15

**Authors:** Guohong Gao, Zhilong Yu, Xiaoyan Zhao, Xinyi Fu, Shengsheng Liu, Shan Liang, Aijun Deng

**Affiliations:** Department of Ophthalmology, Affiliated Hospital of Weifang Medical University, Clinical Medical Institute, Weifang Medical University, Weifang, 261000 Shandong China

**Keywords:** Cancer, Computational biology and bioinformatics, Biomarkers

## Abstract

Cutaneous melanoma could be treated by immunotherapy, which only has limited efficacy on uveal melanoma (UM). UM immunotyping for predicting immunotherapeutic responses and guiding immunotherapy should be better understood. This study identified molecular subtypes and key genetic markers associated with immunotherapy through immunosignature analysis. We screened a 6-immune cell signature simultaneously correlated with UM prognosis. Three immune subtypes (IS) were determined based on the 6-immune cell signature. Overall survival (OS) of IS3 was the longest. Significant differences of linear discriminant analysis (LDA) score were detected among the three IS types. IS3 with the highest LDA score showed a low immunosuppression. IS1 with the lowest LDA score was more immunosuppressive. LDA score was significantly negatively correlated with most immune checkpoint-related genes, and could reflect UM patients’ response to anti-PD1 immunotherapy. Weighted correlation network analysis (WGCNA) identified that salmon, purple, yellow modules were related to IS and screened 6 prognostic genes. Patients with high-expressed NME1 and TMEM255A developed poor prognosis, while those with high-expressed BEX5 and ROPN1 had better prognosis. There was no notable difference in OS between patients with high-expressed LRRN1 and ST13 and those with low-expressed LRRN1 and ST13. NME1, TMEM255A, Bex5 and ROPN1 showed potential prognostic significance in UM.

## Introduction

UM is the most common primary intraocular tumor, occurring mainly in choroid, ciliary body, and iris. Surgical resection, radiation therapy, and enucleation are primary options for treating UM^1^. These therapies can achieve local control of UM, but about half of patients still develop metastasis most often to the liver^2^, and the median OS was only 13.4 months for UM patients with cancer metastasis^3^. Current therapies for managing metastatic UM are systemic chemotherapy and hepato-targeted therapy^4^, however, the response of metastatic UM to systemic chemotherapy is low^5^. Moreover, patients with hepatic metastatic UM benefit limitedly from liver-targeted therapy^6^. Immunotherapy could offer new treatment possibilities for metastatic UM treatment.

Immune checkpoint inhibitors have been successfully used in the treatment of cutaneous melanoma. The study of Caroline Robert et al. showed that the anti-programmed cell death protein 1 (PD-1) antibody pembrolizumab extends progression-free survival and OS of patients with advanced melanoma^7^. Immunotherapy response rates of metastatic patients to anti-PD-1 (nivolumab) combined with anti-cytotoxic T-lymphocyte associated Protein 4 (CTLA-4) (ipilimumab) are as high as 60%^8^. So far, immunotherapy has limited success in metastatic UM^9^. UM immunotherapy remains a clinical challenge for its spatial intra-tumor heterogeneity^10^. Study of new immune strategies may lead to long-term immune responses^11^, which requires in-depth understanding of the complex immune molecular changes in tumor microenvironment and immunological characteristics of UM. Here, a comprehensive immunotyping of UM may be an effective strategy.

Various cancers have been subtyped based on tumor immune signatures or genes, helping guide immunotherapy or prognostic prediction. Robertson et al. reported four subtypes of different molecular characteristics through conducting a comprehensive study using multiple omics^12^, which demonstrated the effectiveness of stratified management and treatment of UM patients. Jingkai Liu et al. determined three immune subtypes with different immune features and prognosis by clustering five immune signature gene sets^13^. Cholangiocarcinoma patients could be stratified into two immune subtypes that are characterized by differential molecular, cellular and clinical features^14^. Based on gene expression, diffuse glioma has also been classified into three immune subtypes, which demonstrated significant differences in immune infiltration, immune checkpoint gene expression, and clinical characteristics^15^. These findings suggested that immunotyping may have great potential in immunotherapy.

In this study, we clustered UM samples in The Cancer Genome Atlas (TCGA, https://portal.gdc.cancer.gov/) dataset according to the 6-immune cell signature associated with patients’ prognosis, identified three ISs, and evaluated the relationship between the three subtypes and tumor microenvironment. The IS-related modules were screened after the construction of co-expression network, and the genes that could effectively predict the prognosis of UM were screened. These results may improve our understanding of UM immunotherapy.

## Results

### Three ISs were identified by consensus clustering

**Figure 1:**
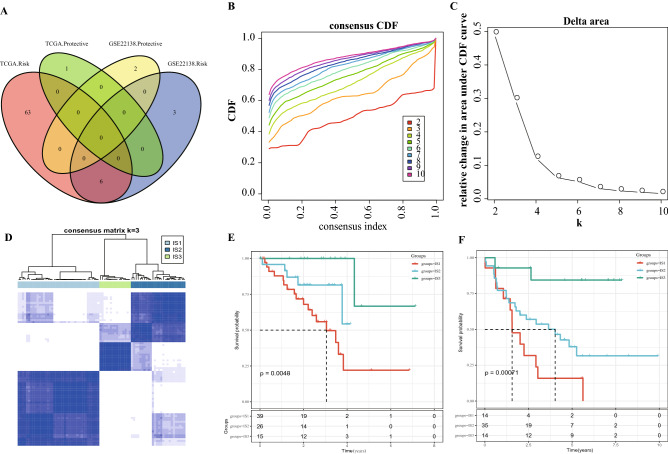
Consensus clustering identified three ISs of UM. (**A**) Venn diagram of intersection of immune cell signature significantly related to OS in patients with UM from TCGA and GSE22138 cohort. (**B**) CDF curves of TCGA cohort samples. (**C**) CDF Delta area curve of TCGA cohort samples (Delta area curve of consensus clustering, indicating the relative change in area under the CDF curve for each category number k compared with k − 1. The horizontal axis represents the category number k and the vertical axis represents the relative change in area under CDF curve). (**D**) The sample clustering heat map at k = 3. (**E**,**F**) Kaplan–Meier survival curves of patients with different ISs in the GSE22138 cohort.

In the TCGA cohort, a total of 70 out of 119 immune cell signatures were related to UM prognosis. There were 11 immune cell signatures significantly related to OS of UM patients in the GSE22138 cohort. To avoid difference caused by a single immune cell signature in different cohorts, 6 common immune cell signatures shared by the two cohorts were taken for subsequent analysis (Fig. 1A). 80 UM samples were clustered, and the cumulative distribution function (CDF) was used to determine the optimal number of clustering. We found that the clustering result was relatively stable when Cluster = 3 (Fig. 1B–D). Next, UM was divided into three ISs. Kaplan–Meier analysis on the correlation between the three ISs and OS showed significant differences in OS of the three ISs in both TCGA cohort and GSE22138 cohort. Noticeably, the OS of the IS3 subtype was longer when compared with other two ISs (Fig. 1E,F). These results indicated that UM samples from different clusters can be distinguished based on immune cell signatures.

### Genetic mutations in different IS

**Figure 2:**
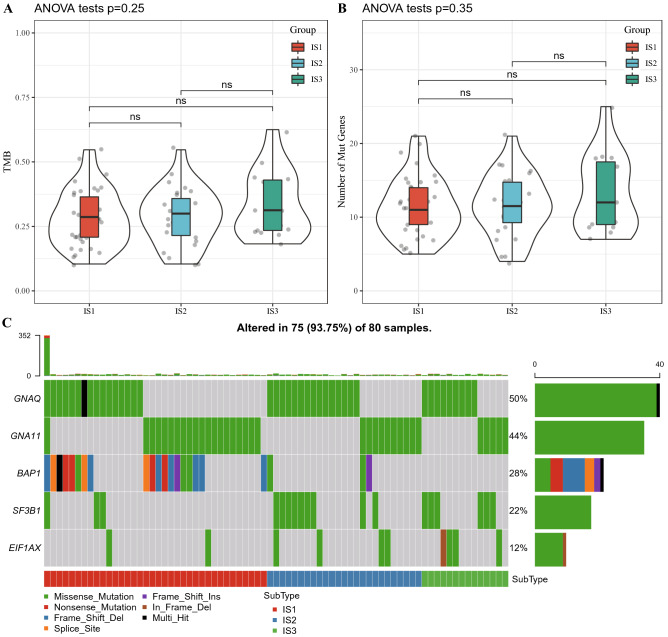
Genetic mutations in different ISs. (**A**) TMB differences between the three ISs. (**B**) Difference analysis was used to compare the number of gene mutations in the three IS samples. (**C**) Mutation characteristics of the top5 significantly mutated genes in each IS samples. *P < 0.05, **P < 0.01. NS: not significant.

Tumor mutational burden (TMB) and gene mutation frequency in the three ISs were analyzed, and there was no significant difference in TMB among the three ISs (Fig. 2A,B). The frequency of mutant genes in different IS was counted and ranked from high to low by mutation frequency. The mutation characteristics of Top 5 genes in each subtype are shown in Fig. 2C. The mutation frequency of GNAQ was as high as 50%, followed by GNA11 (44%) and BAP1 (28%) as the second and the third gene with a higher mutation rate, respectively.

### Associations of IS, classical markers, and immune checkpoints of chemotherapy-induced immune response

**Figure 3:**
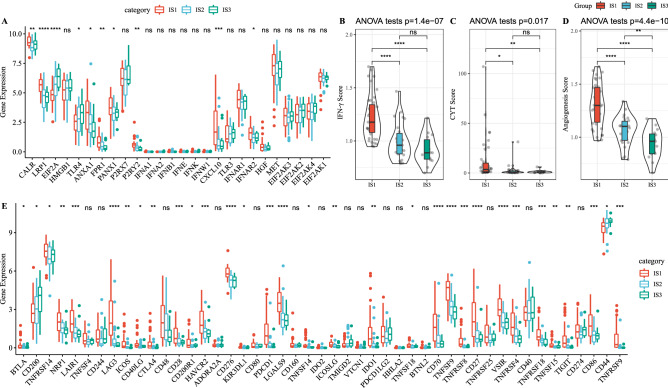
Associations between ISs and classical markers and immune checkpoints of chemotherapy-induced immune response. (**A**) Difference in expression of classic markers of chemotherapy-induced immune response in the three IS samples. (**B**) Differences in IFN-γ scores among the three ISs. (**C**) CYT score differences between the three ISs samples. (**D**) Differences in angiogenesis score among the three ISs. (**E**) The expression of 47 immune checkpoint-related genes differed in the three ISs. *P < 0.05; **P < 0.01; ***P < 0.001; ****P < 0.0001; Ns: not significant.

We selected 26 genes with expression levels for differential analysis to analyze expression differences in classic markers of chemotherapy-induced immune response in the three IS samples. In the three IS samples, there were 10 genes showing significant expression differences (Fig. 3A), suggesting that UM samples from different IS may have different responses to chemotherapy. We also calculated the IFN-γ score of each patient using SSGSEA according to the TH1/IFN-γ gene signature^16^. Here, we found that the IFN-γ score was notably different in the three ISs. Specifically, the IS1 subgroup had the highest IFN-γ score, while the IS3 subgroup had the lowest IFN-γ score (Fig. 3B). Cytolytic activity (CYT) in patients with different IS was determined based on the mean expression levels of two key cytolytic effectors (GZMA and PRF1), and the results demonstrated significant differences in CYT scores among the three IS samples (Fig. 3C). Angiogenic signature used to evaluate the angiogenic scores of patients with various subtypes of UM showed that there were significant differences in angiogenesis scores among the three ISs (score in IS1 > IS2 > IS3) (Fig. 3D). Moreover, we also studied the expression differences of 47 immune checkpoint-related genes in the three ISs. From Fig. 3E, it can been seen that 32 genes were differentially expressed in the three ISs.

**Figure 4:**
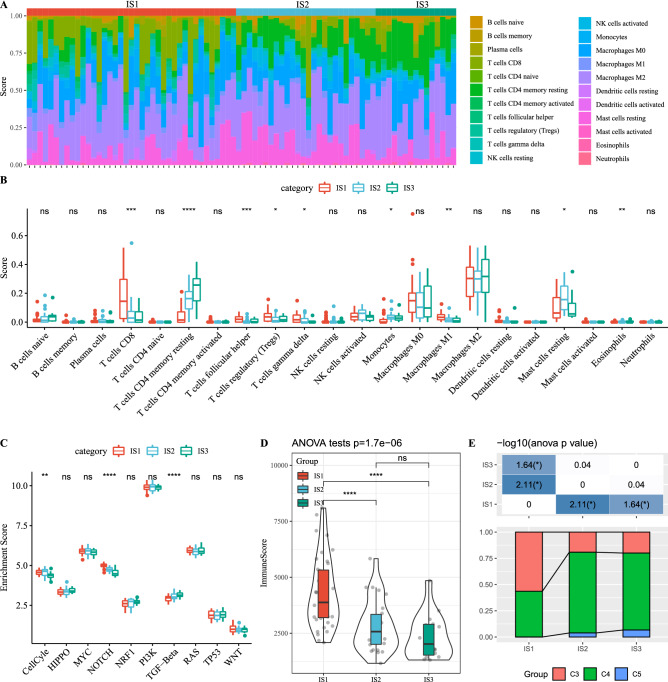
Association between IS and tumor immune microenvironment. (**A**) Heatmap of the degree of immune infiltration of 22 kinds of immune cells in different IS samples. (**B**) Box plots showing the relationships between IS and immune cell infiltrations. (**C**) Enrichment of three ISs in 10 oncogenic pathways. (**D**) Differences in immune scores between the three IS samples. (**E**) Comparison of the distribution of immune subtypes in different ISs. *P < 0.05; **P < 0.01; ***P < 0.001; ****P < 0.0001; Ns: not significant.

The relationship between the three ISs and tumor microenvironment was also analyzed, and the scores of 22 immune cells in different IS samples were assessed by CIBERSORT. The analytical results in Fig. 4A,B showed that among the three subtypes of samples, CD8 T cells, resting memory CD4 T cells, follicular helper T cells, regulatory T cells, gamma delta T cells, monocytes, M1 macrophages, resting mast cells and eosinophils were significantly different. Through analyzing the enrichment of the three IS types in 10 oncogenic pathways, significant differences in Cell Cycle, Notch, and TGF-β pathway enrichment scores of the three IS samples were confirmed. The enrichment scores of Cell Cycle and Notch in IS1 were significantly higher than that in IS3, and the enrichment scores of TGF-β pathway in IS1 were sharply lower than that in IS3 (Fig. 4C). After calculating the immune score of each IS sample, a significant correlation between immune score and IS was identified, and the immune score was IS1 > IS2 > IS3 (Fig. 4D). Furthermore, we analyzed the relationships between the three ISs and the six immunosubtypes of pan-cancer, and the data revealed significant differences in the pan-cancer immunotyping in IS1, IS2, and IS3. We also noted that C3 with a better prognosis accounted for a large proportion of the immunomolecular subtype IS1 defined by this study (Fig. 4E). This suggested that these three subtypes were supplementary to the 6 pan-cancer immunosubtypes.

### Construction of LDA score

**Figure 5:**
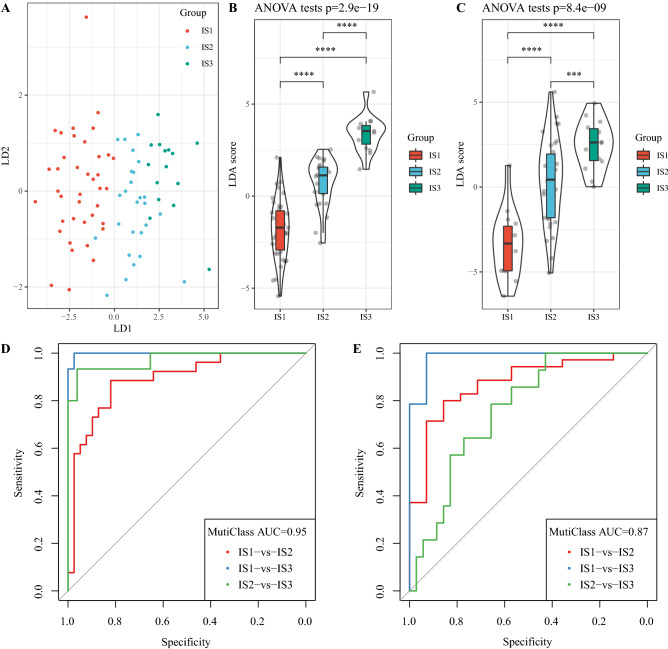
The LDA score can be used to measure the different immune characteristics of patients. (**A**) Relationship between the first 2 features of LDA score and ISs. (**B**) LDA score difference of different IS samples in TCGA data set. (**C**) LDA score differences of three ISs in GSE22138 data set. (**D**) ROC curve of LDA score in TCGA data set. (**E**) ROC curve of LDA score in GSE22138 data set. ***P < 0.001; ****P < 0.0001.

Z transform of the 6-immune cell signature was performed, and LDA was used to establish subtype classification feature index. The first two features of the model could correctly distinguish samples of different subtypes (Fig. 5A). Based on the LDA model, we calculated the LDA score of each patient in the TCGA data set, and found significant differences in LDA score of the three ISs (Fig. 5B). Receiver Operating Characteristic (ROC) was applied to determine the classification performance of LDA score in different subtypes, and the combined predicted AUC of multiple categories was 0.95. In addition, the LDA scores of GSE22138 dataset samples classified by IS showed similar results to TCGA dataset. LDA scores of different subtypes were significantly different (Fig. 5C). ROC analysis demonstrated that the combined AUC was 0.87 (Fig. 5D,E). This suggested that LDA score can be used to measure different immune characteristics of patients. The LDA score of IS3 was the highest, indicating a low immunosuppression. IS1 had the lowest LDA score and was more immunosuppressive.

### Relationship between LDA score and immunotherapy

**Figure 6:**
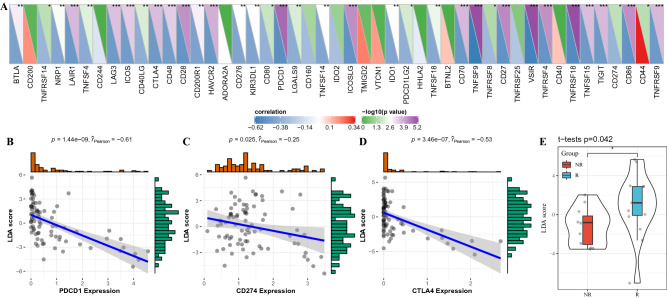
Relationship between LDA score and immunotherapy. (**A**) Relationship between LDA score and immune checkpoint. (**B**) Correlation between LDA score and PDCD1. (**C**) Correlation between LDA score and CD274 expression. (**D**) Correlation between LDA score and CTLA4 expression. (**E**) The LDA score of patients who responded to PD-1-blocking immunotherapy was significantly higher than that of patients did not respond to immunotherapy. *P < 0.05; **P < 0.01; ***P < 0.001.

To explore the relationship between LDA score and immunotherapy, the relationship between LDA score and immunoexamination points was first analyzed. Pearson correlation coefficient was employed to calculate the correlation between LDA score and the expression of related genes at 47 immune examination points. We found that LDA score was significantly negatively correlated with most immune checkpoint-related genes (Fig. 6A). Figure 6B–D showed the correlation analysis results of LDA score and the expression of PD-L1, PD1, CTLA4. The results presented significant differences in the LDA scores of patients with different immune response states before anti-PD-1 treatment, and the LDA score of patients responsive to PD-1-blocking immunotherapy was significantly higher than that of patients did not respond to immunotherapy (Fig. 6E). Therefore, the immunotherapy response status of UM patients can be evaluated according to LDA score.

### Construction of co-expression network and identification of IS-related modules

**Figure 7:**
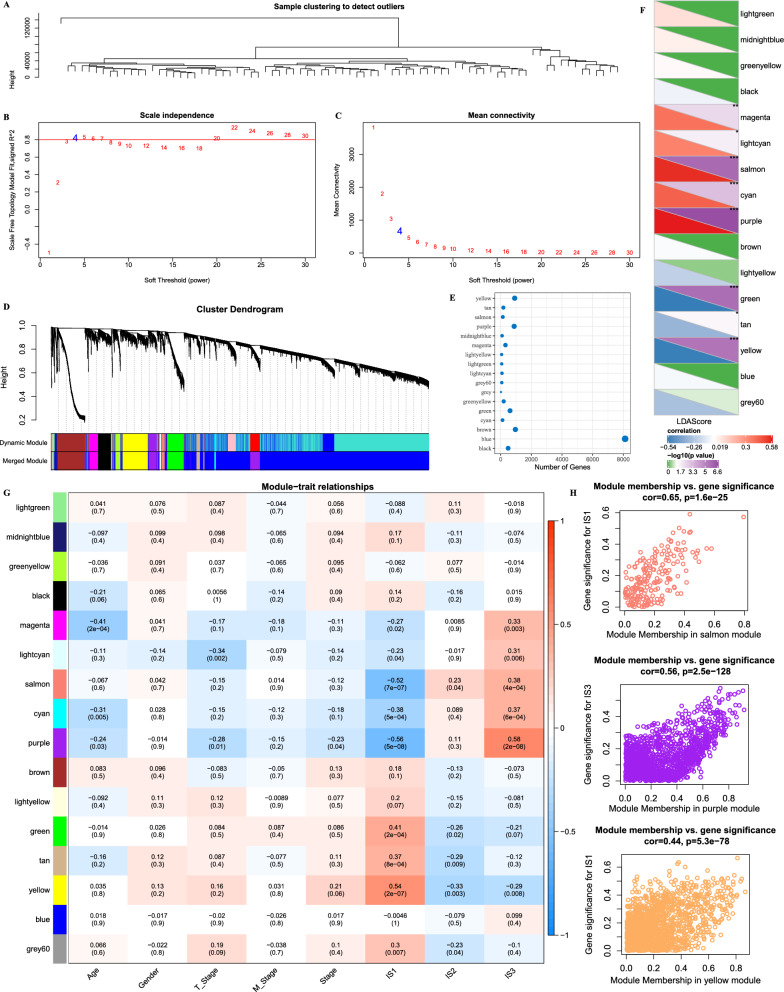
Construction of co-expression network and identification of IS-related modules. (**A**) Clustering tree of UM samples in TCGA. (**B**,**C**) Analysis of the scale-free fit index and mean connectivity for various soft-thresholding powers (β). (**D**) 17 modules were identified by unsupervised clustering in WGCNA. (**E**) Transcript number statistics of each module. (**F**) Correlation between 17 modules and LDA score. (**G**) Correlation analysis between each module and clinical information. (**H**) Scatter plots of module eigengenes in the salmon, purple and yellow modules. *P < 0.05; **P < 0.01; ***P < 0.001; ****P < 0.0001.

The UM samples in TCGA were clustered to calculate the network topology of β from 1 to 30, and a scale-free network was developed based on β = 4 (Fig. 7A–C). The dynamic shearing method was used to show gene modules, and eigengenes of each module were calculated. After conducting cluster analysis, modules close to each other were merged into new modules (Height = 0.25, DeepSplit = 2, MinmoduleSize = 30). Finally, a total of 17 modules were obtained (Fig. 7D), and each module had different number of genes (Fig. 7E). The correlation analysis between the feature vectors of 17 modules and LDA score revealed that 8 out of 17 modules were significantly correlated with LDA score (Fig. 7F). In addition, the correlation between the module and clinical characteristics (age, sex, T Stage, M Stage, Stage, IS1, IS2, and IS3) was analyzed, and we observed that the yellow or salmon module was significantly correlated with IS1, IS2, and IS3, and that the yellow module was positively correlated with IS1 and significantly negatively correlated with IS2 and IS3. Salmon module was negatively correlated with IS1, and significantly positively correlated with IS2 and IS3 (Fig. 7G). The correlation analysis results of genetic significance (GS) and module significance (MM) in salmon, purple and yellow modules are shown in Fig. 7H.

### Identification of prognosis-related genes in the module

**Figure 8:**
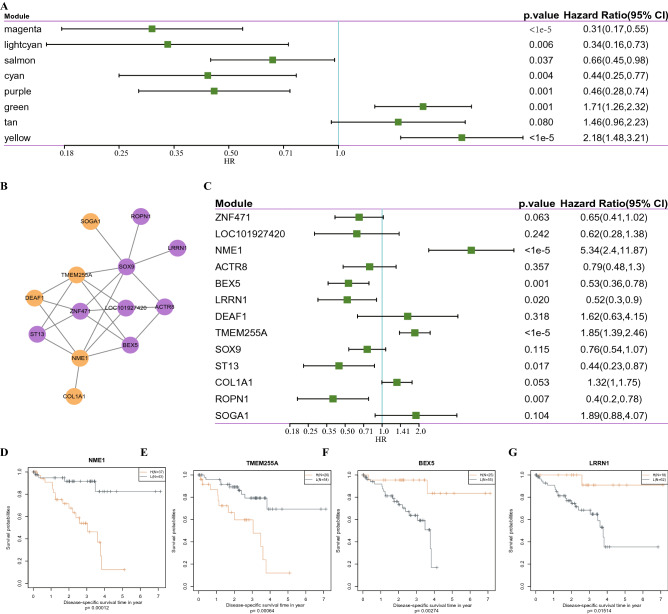
Identification of prognosis- related genes in the module. (**A**) Univariate Cox analysis of the correlation between modules related to LDA score and OS. (**B**) Potential genetic markers associated with LDA score. (**C**) The correlation between 13 genes and UM prognosis was analyzed by univariate Cox analysis. (**D**–**G**) Kaplan–Meier curves of NME1, TMEM255A, BEX5, and ROPN1 expression in UM patients.

To identify genes associated with UM prognosis, the correlation between modules and prognosis was analyzed. From the univariate Cox analysis, the salmon, purple and yellow modules were significantly correlated with the prognosis. Salmon and purple modules were protective of UM, while yellow module was the risk module (Fig. 8A). A total of 13 genes with a co-expression weight greater than 0.77 were identified from these three modules, here 8 genes were in purple module and the rest 5 genes were in yellow module (Fig. 8B). The correlation between these 13 genes and UM prognosis was further analyzed, and 6 genes (NME1, Bex5, LRRN1, TMEM255A, ST13, ROPN1) were found to be significantly correlated with prognosis (Fig. 8C). Based on survival analysis, patients with high expression of NME1 and TMEM255A tended to develop a poor prognosis (Fig. 8D,E), while those with high expression of BEX5 and ROPN1 showed a better prognosis (Fig. 8F,G). There was no significant difference in OS between patients with high expression of LRRN1 or ST13 and patients with low LRRN1 or ST13 expression (Supplementary Fig. S1). Furthermore, we obtained the expression profile data set of patients before treatment of anti-PD1 from previous studies^17^, and analyzed the differences of NME1, TMEM255A, BEX5 and ROPN1 expression profile under different response states before treatment. It could be observed that NME1, BEX5 and ROPN1 were significantly overexpressed in the immune response group, while TMEM255A was significantly low-expressed in the immune response group (Supplementary Fig. S2). Therefore, NME1, TMEM255A, BEX5 and ROPN1 were confirmed as prognostic markers of UM.

## Discussion

TNM staging has long been one of the most important indicators to predict cancer patients’ prognosis. However, cancers shows unique clinical characteristics, patient prognosis, and treatment response due to high heterogeneous nature^18^. Emerging reports demonstrates a stronger prognostic significance of immunoclassification than TNM classification^19^. Identification of UM subtypes facilitate biological research and deciding personalized treatment for UM patients. At present, the molecular subtypes of UM could be classified according to the specific characteristics of UM. Studies have identified two molecular subtypes of UM based on the expression of m6A RNA methylation modulator^20^. Berylroyer-Bertrand et al*.* performed unsupervised hierarchical clustering on UM samples according to copy number variation, and determined four major subgroups associated with metastatic and mutation status^21^. In addition, previous study screened three different ISs with prognostic significance for UM^22^. But few studies have identified UM molecular subtypes in terms of immune cell signatures.

In this study, we determined the ISs of UM through immune cell signatures. Firstly, a 6-immune cell signature correlated with UM prognosis was screened from 119 immune cells in the TCGA and GSE22138 cohorts. Based on the 6-immune cell signature, three ISs showing significant OS differences (IS3 > IS2 > IS1) were identified. Previous studies have detected frequent oncogenic mutations of GNAQ and GNA11 in primary UM^23,24^. Here, we also found that the mutation frequency of GNAQ and GNA11 in the three ISs was as high as 50% and 44%, respectively, which was consistent with previous studies. In addition, 10 out of the 26 classical markers of chemotherapy-induced immune responses showed significant expression differences among the three IS types. IFN-γ plays a key role in activating cellular immunity and stimulating anti-tumor immune responses^25^. There were also showed significant differences in IFN-γ scores of the three ISs. The study of Florian Castet et al*.* demonstrated that the enrichment of pro-angiogenic factors is related to a poor prognosis of UM^26^. CYT has also been found to be related to counter-regulatory immune responses and a better prognosis^27^. Thus, we evaluated the angiogenesis score and CYT score among the three IS samples, and detected great differences in the angiogenesis score and CYT score among the three IS samples (IS1 > IS2 > IS3).

Adaptive immune cells and innate immune cells in tumor microenvironment are contributor factors to tumor progression^28^. Great differences were detected in the infiltration scores of CD8 T cells, resting memory CD4 T cells, follicular helper T cells, regulatory T cells, gamma delta T cells, monocytes, M1 macrophages, resting mast cells and eosinophils in the three IS samples. The immune score of IS1 was significantly higher than that of IS2 and IS3.The LDA score was developed to evaluate different immune characteristics of UM patients, and the LDA score of IS3 was the highest, indicating a low degree of immunosuppression. IS1 had the lowest LDA score and was therefore more immunosuppressive.

Immune checkpoints are key modulators of immune system function^29^. In our study, the expression of immune checkpoint-related genes of three ISs was analyzed, surprisingly, we found that the expression of T cell failure markers such as LAG3, HA VCR2, CTLA4, PDCD1, CD274^30–32^ was significantly higher in IS1 than in IS3. However, IS1 had a high CYT score but the worst prognosis when compared with the other two categories, suggesting that T cell exhaustion may exist in IS1. Current immunotherapies targeting various immune checkpoints include CTLA4, PD-1, and programmed death ligand 1 (PD-L1)^33^. Our LDA score was significantly negatively correlated with most immune checkpoint-related genes, such as CTLA4, PD-1, and PDL1. Moreover, LDA score of patients with different immune response states before anti-PD-1 treatment also showed great differences. This indicated that LDA score can be used to determine the response of UM patients to immunotherapy, which may guide further immunotherapy.

To better analyze the role of immunotyping in prognosis, WGCNA was performed to three modules significantly associated with LDA score and prognosis, and we screened four potential prognostic markers of UM. Whether NME1 was indicative of favorable or unfavorable prognosis still remained unclear^34^. In neuroblastoma, high expression of NME1 is associated with poor prognosis of patients^35^, but NME1 is tumor-suppressive in many human cancers, including in breast cancer, melanoma and lung cancer^36–38^. Here, we found that patients with high expression of NME1 and TMEM255A tended to develop a poor prognosis, which may be explained by the heterogeneity of NME1 in the melanoma domain^37^. The role of TMEM255a in tumor has not been reported. Moreover, our study also showed that patients with high expression of BEX5 and ROPN1 had better prognosis. The roles of these two genes have not been reported in cancer.

In summary, based on immune cell signatures, we identified three ISs showing significant heterogeneity in tumor microenvironment, immunotherapy response status, and LDA score. Four genes identified by WGCNA were associated with the prognosis of UM. Detailed immunotyping of UM is important for predicting patient survival and may help identify patients who would benefit from anti-tumor immunotherapy.

## Methods

### Data source and preprocessing

The study was approved by Ethical Committee of the Affiliated Hospital of Weifang Medical University. Informed consent was given by patients. We confirmed that all methodological researches were performed in accordance with relevant guidelines and regulations. The clinicopathological data and RNA-seq data of 80 UM samples were acquired from TCGA. ENSG was matched to GeneSymbol, and a total of 25,483 gene profiles were obtained. GSE22138 microarray data from Gene Expression Omnibus (GEO) database contained 63 UM samples. A total of 23,520 gene expression profiles were acquired after probe and gene matching. Median was taken when multiple probes matched to one gene, and deletion was performed when probes matched to multiple genes appeared.

### Immune cell signatures analysis

Sample enrichment score was calculated using the 119 tumor microenvironment-associated immune cell signatures in the R packet IOBR. Univariate survival analysis screened and intersected UM prognostic signatures in TCGA and GSE22138 cohorts.

### Identification of immune cell signature-associated subtypes

ConsensusClusterPlus was used to construct a consistency matrix, and the samples in TCGA dataset were classified through clustering^39^. 500 Bootstraps were carried out using Partitioning Around MEDOI (PAM) algorithm. Euclidean was used as the measurement distance. The clustering number "k" was set between 2 and 10, and the optimal number of clustering was determined by the cumulative distribution function (CDF).

### TMB analysis of IS

Mutation data sets processed by mutect2 software were downloaded to calculate TMB and analyze TMB differences of the IS. Then the frequency of gene mutation in different immunotyping samples was counted.

### Associations between IS and tumor microenvironment

The absolute immune cell score of each sample was calculated by the genetic signatures of 22 immune cell types by CIBERSORT^40^, and the differences in the scores of these immune cells among different ISs were also counted. In addition, the enrichment of ISs in 10 typical pathways (cell cycle, Hippo, Myc, Notch, Nrf2, PI-3-Kinase/Akt, RTK-RAS, TGFβ signaling, p53 and β-catenin/Wnt) was analyzed and compared with 10 typical pathways^41^.

### LDA

LDA, which is a widely used method for pattern classification, could well present small number of features for prediction^42^. Considering that different ISs have different molecular characteristics, to better quantify the immune characteristics of patients in different sample cohorts, we used LDA to establish subtype classification feature index (a LDA score), and evaluated the classification performance of the LDA score in different subtypes by ROC analysis.

### Establishment of weighted co-expression network

The TCGA expression profile data set was used to screen genes with MAD greater than 50% as the gene expression profile, subsequently co-expression network of these genes was constructed by WGCNA^41^. The samples were clustered to identify co-expression modules. Appropriate soft threshold parameter was determined to ensure a scale-free nature of the network. Next, the presentation matrix was transformed into an adjacency matrix, which as further converted to a topological overlap matrix (TOM). Average-linkage hierarchical clustering method was used for gene clustering, and the minimum number of genes in each gene network module was set to 30, according to the standard of hybrid dynamic shear tree.

### Identification of prognosis-related modules and genes

To screen the LDA score-related modules, the correlation between gene vectors of modules and LDA score, between modules and clinical phenotypes were analyzed. Then the prognosis-related modules were further screened to extract genes for investigating their correlation with UM prognosis.

## Supplementary Information

Supplementary Figures.

## Data Availability

The datasets generated during and/or analysed during the current study are available in the in thee [GSE22138] repository, (https://www.ncbi.nlm.nih.gov/geo/query/acc.cgi?acc=GSE22138).
