# Supplementary material for: Immune classification and identification of prognostic genes for uveal melanoma based on six immune cell signatures
Source: Sci Rep. 2021 Nov 15;11:22244. doi: 10.1038/s41598-021-01627-2 (PMC8593069; doi:10.1038/s41598-021-01627-2)
Supplement: Supplementary file 1 — Supplementary Figures. [file 41598_2021_1627_MOESM1_ESM.pdf]

Identification of immune classification and prognostic genes for uveal melanoma based on six immune cell signatures

Guohong Gao[1*a], Zhilong Yu [1a], Xiaoyan Zhao[1], Xinyi Fu[1], Shengsheng Liu[1], Shan Liang[1], Aijun Deng[1]

[1] Department of Ophthalmology, Affiliated Hospital of Weifang Medical University, Clinical Medical Institute, Weifang Medical University,Weifang,Shandong,China, 261000.

***Corresponding author**

Guohong Gao, Department of Ophthalmology, Affiliated Hospital of Weifang Medical University, Clinical Medical Institute, Weifang Medical University,Weifang,Shandong,China, 261000, eyeggh@163.com.

[a] equal contribution

Supplementary material captions

Figure S1 Prognosis Kaplan-Meier curves based on ST13 or ROPN1 gene expression grouping.

Figure S2 Expression differences of 4 genes in immunotherapy group.

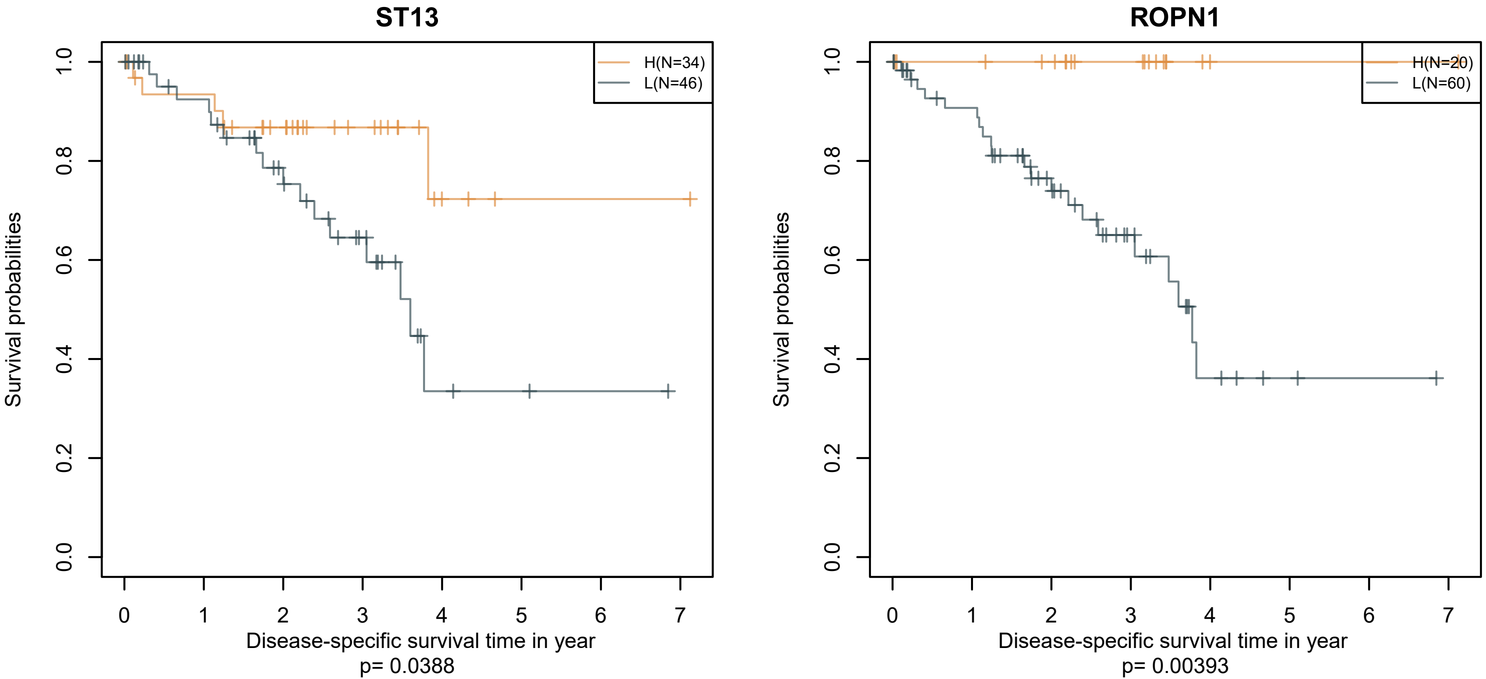
ST13
H(N=34)
L(N=46)
Survival probabilities
0.0
0.2
0.4
0.6
0.8
1.0
0
1
2
3
4
5
6
7
Disease-specific survival time in year
p= 0.0388
ROPN1
H(N=20)
L(N=60)
Survival probabilities
0.0
0.2
0.4
0.6
0.8
1.0
0
1
2
3
4
5
6
7
Disease-specific survival time in year
p= 0.00393

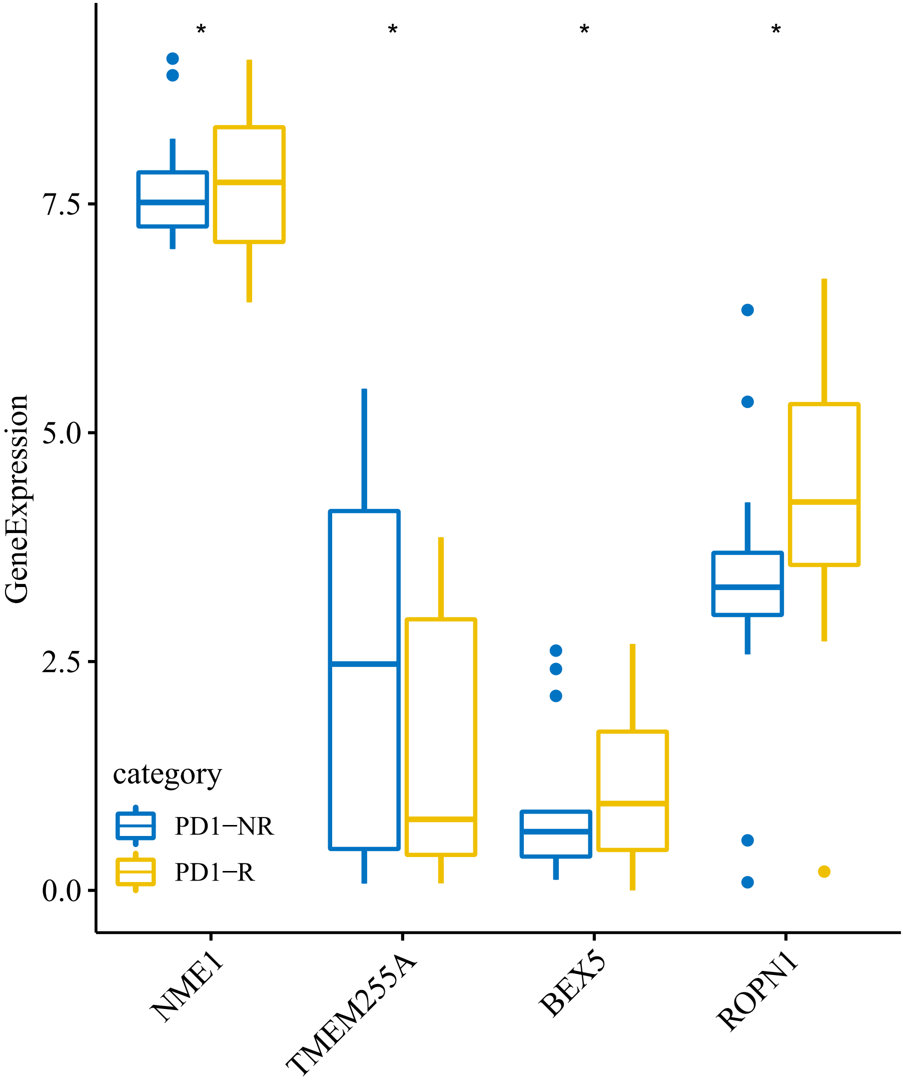

*
*
*
*
GeneExpression
7.5
5.0
2.5
0.0
category
PD1−NR
PD1−R
NME1
TMEM255A
BEX5
ROPN1
